# Personal Characteristics Effects on Validation of Self-reported Type 2 Diabetes From a Cross-sectional Survey Among Chinese Adults

**DOI:** 10.2188/jea.JE20190178

**Published:** 2020-11-05

**Authors:** Hong-Lan Li, Jie Fang, Long-Gang Zhao, Da-Ke Liu, Jing Wang, Li-Hua Han, Yong-Bing Xiang

**Affiliations:** State Key Laboratory of Oncogenes and Related Genes & Department of Epidemiology, Shanghai Cancer Institute, Renji Hospital, Shanghai Jiaotong University School of Medicine, Shanghai, China

**Keywords:** validation study, self-report, type 2 diabetes, personal characteristics

## Abstract

**Background:**

The objective was to evaluate the effects of personal characteristics on the validation of self-reported type 2 diabetes among Chinese adults in urban Shanghai.

**Methods:**

During 2015 through 2016, 4,322 participants were recruited in this validation study. We considered the criteria of diabetes verification to use the laboratory assays of fasting plasma glucose (FPG), glycated hemoglobin (HbA1c), or self-reported use of diabetic medication.

**Results:**

When taking diabetic medication or FPG ≥7.0 mmol/L was as identified diabetes, the measurements of sensitivity, specificity, positive predictive value (PPV), negative predictive value (NPV), and Kappa value of self-reported diabetes were 72.0%, 99.2%, 95.1%, 93.9%, and 0.78, respectively. If an additional HbA1c test was used for 708 subjects (aged <65 years), slightly lower values of sensitivity, NPV, and Kappa were observed. More potential diabetes cases were found compared to only using FPG. Subjects who were female, older, or had a family history of diabetes had sensitivity over 75% and excellent Kappa over 0.8, while the sensitivity and Kappa of opposite groups had poorer values. Specificity, PPV, and NPV were similar among groups with different demographic or disease characteristics. The prevalence of type 2 diabetes was 19.3% in the study (14.1% diagnosed diabetes, 5.2% undiagnosed diabetes). About 26.2% of subjects were pre-diabetic. Additional HbA1c test indicated an increased prevalence of undiagnosed diabetes and pre-diabetes.

**Conclusions:**

Findings support self-reported diabetes is sufficiently valid to be used in large-scale, population-based epidemiologic studies. Participants with different characteristics may have different indicators in terms of validation, such as age, gender, and family history of diabetes in first-degree relatives.

## INTRODUCTION

According to the International Diabetes Federation (IDF), 425 million adults (aged 20–79 years) had diabetes in 2017, and the projected number will rise to 629 million by 2045. In China, the diabetes prevalence adjusted to the world population is 9.7%, well above the global prevalence of 8.8%. China has the highest number of people with diabetes, with over 114 million adults affected in 2017, and the number will reach 120 million in 2045. Approximately 4.0 million people aged between 20 and 79 years died of diabetes in 2017 in the world, which accounted for 10.7% of global all-cause mortality among people in this age group.^1^^,^^2^ Diabetes is also associated with an increased risk of death from other causes, which are substantially more than that directly coded to diabetes.^3^

It is important in epidemiologic studies to confirm diabetes diagnosis. The prevalence of diabetes in the population is commonly gathered using questionnaires. A self-reported questionnaire is an important and convenient tool; sometimes, it is the only feasible way to obtain information on subject health status in epidemiologic surveys when laboratory assays were absent but diabetes status was available. The accuracy of self-reported diabetes will affect the results in diabetes-related epidemiologic studies. As a consequence, it is important to assess the validity of that information. Medical records, administrative databases, and measurement of glucose are often used as gold standards in validation studies.^4^^–^^16^ Many population-based cohort studies conducted in developed countries have addressed the validity of self-reported diseases, such as studies in America,^4^^,^^9^^,^^13^^,^^14^^,^^17^ Australia,^5^ the Netherlands,^10^ Britain,^18^ Japan,^6^^,^^15^^,^^19^^,^^20^ and Singapore.^21^ Diabetes is considered to have clear diagnostic criteria, requires ongoing management, and is more reliably reported than other chronic diseases.^7^^,^^11^^,^^22^ Most of these studies rely on comparison to medical records and confirmations of self-reported diabetes.^4^^,^^8^^,^^11^^,^^13^^,^^17^^–^^19^^,^^21^ Some studies compared to glucose-related measurements.^6^^,^^9^^,^^10^^,^^12^^,^^14^^–^^16^^,^^20^ The characteristics of the study participants, such as age, gender, and education contribute to awareness of diabetes.^6^^,^^11^

In the present analysis, we evaluated the validity of type 2 diabetes self-reports in a community-based survey in urban Shanghai by comparing with glucose-related measurements.

## METHODS

### Study participants

From 2015 through 2016, a community-based cross-sectional survey was initiated as a validation study of diabetes self-reports. In two study communities, a total of 4,322 subjects (1,681 women and 2,641 men) agreed to have the health checkups and additional self-reported information on diabetes collected just before the checkup. All the participants of the present study are from the Shanghai Women’s Health Study and the Shanghai Men’s Health Study, two population-based prospective cohorts conducted in urban Shanghai, China.^23^^,^^24^ The inquiry about diabetes was set up like that in the follow-up surveys in these two cohorts.

### Assessment of self-reports

Self-reported diabetes status was assessed by baseline and follow-up questionnaires, which included the collection of demographic characteristics for all study participants. The inquiries about diabetes were set up and contained the following questions: (a) We would like to know whether you have been diagnosed with diabetes by a physician (yes or no); (b) Date of diagnosis (year & month); (c) Hospital of diagnosis; (d) Have you ever taken fasting blood glucose test? (yes, no, or unknown); (e) Fasting blood glucose ≥7 mmol/L? (yes or no); (f) Number of occurrences of elevated fasting plasma glucose (1 time or ≥2 times); (g) Blood glucose 2 hours after meal ≥11.1 mmol/L? (yes or no); (h) Number of occurrences of elevated blood glucose 2 hours after meal (1 time or ≥2 times); (i) Any symptoms of diabetes, such as increased thirst, increased hunger, frequent urination, or unexplained weight loss? (yes or no); (j) Ever used hypoglycemic drugs or insulin? (yes or no); and (k) Date started taking medicine (year & month). We defined the diabetes cases whose diagnosis ages were younger than 20 years as the potential patients with type 1 diabetes. In this study, all diabetes patients’ diagnosis ages were over 20 years and were considered as type 2 diabetes.

### Laboratory tests

Laboratory assay of FPG was obtained at the checkup in the community health centers. HbA1c was additionally performed in one community for all subjects (403 females and 305 males) less than 65 years old who consented. After an overnight fast of at least 10 hours, venous blood samples were collected in tubes that contained EDTA for FPG or HbA1c tests in the community health centers. Before blood collection, the nurses asked subjects whether he/she had taken diabetic medication in the past 24 hours.

For validation of prevalent self-reported diabetes, we compared self-report with a reference definition: a FPG ≥7.0 mmol/L (126 mg/dL) or HbA1c ≥6.5% or treatment with hypoglycemic medications. FPG range of 5.6–6.9 mmol/L or HbA1c range of 5.7–6.4% was used as identifying individuals with pre-diabetes.^25^

### Statistical analysis

Measures of concordance to examine the validity of self-reported diabetes were used in this study, including statistics of sensitivity, specificity, positive predictive value (PPV), negative predictive value (NPV), and Kappa coefficient. PPV was calculated as the number of subjects who reported diabetes and this diagnosis was confirmed by the glucose related measurement or treatment with hypoglycemic medications (true positives) divided by the total number of persons who reported diabetes (total positives). In the same way, NPV was calculated as the number of subjects who reported no diabetes and this diagnosis was confirmed by the glucose related measurement (true negatives) divided by the total number of persons who reported no diabetes (total negatives). Kappa coefficients were calculated to determine the chance corrected agreement between self-reported diabetes of questionnaire data and the glucose-related measurement.^11^^,^^26^ A kappa value of <0.40 was considered poor-to-fair agreement, 0.41–0.60 was considered moderate agreement, 0.61–0.80 was considered substantial agreement, and 0.81–1.00 was considered excellent agreement, as suggested by Landis and Koch.^11^^,^^27^

A two-sided α level of 0.05 was considered statistically significant. The 95% confidence intervals (CIs) for the results were determined by the binomial exact method using SAS software, version 9.2 (SAS Institute, Inc., Cary, North Carolina, USA).

The study was approved by the Institutional Review Boards of the Shanghai Cancer Institute and conducted in accordance with the Declaration of Helsinki Principles. All participants provided written informed consent.

## RESULTS

### General characteristics

General characteristics of subjects in this validation study are presented in Table 1. The average age at baseline of these subjects was 52.8 (standard deviation [SD], 8.23) years old and average body mass index (BMI) was 24.05 (SD, 3.09) kg/m^2^. Compared to those who did not self-report diabetes, the subjects who self-reported diabetes were more likely to be men, older, and ever-smokers and had lower education level, higher BMI, and more family history of diabetes among first-degree relatives.

**Table 1.** Characteristics of subjects in validation study of self-reported type 2 diabetes in Shanghai (*n* = 4,322)

	No self-reported diabetes (*n* = 3,714)		Self-reported diabetes (*n* = 608)		2-sided *P*^a^
	
	number	(%)	number	(%)	
Age at baseline, years					
<60	3,022	(81.4)	464	(76.3)	0.004
≥60	692	(18.6)	144	(23.7)	
Gender					
Male	2,235	(60.2)	406	(66.8)	0.002
Female	1,479	(39.8)	202	(33.2)	
Educational level					
Middle school and below	1,874	(50.5)	336	(55.3)	0.030
High school and above	1,835	(49.5)	272	(44.7)	
BMI, kg/m^2^					
<28	3,415	(92.0)	471	(77.5)	<0.001
≥28	298	(8.0)	137	(22.5)	
Ever smoker					
No	2,174	(58.5)	321	(52.8)	0.008
Yes	1,540	(41.5)	287	(47.2)	
Ever drinker					
No	2,880	(77.5)	480	(78.9)	0.440
Yes	834	(22.5)	128	(21.1)	
Have a family history of diabetes in first-degree relatives					
No	3,002	(81.7)	399	(66.5)	<0.001
Yes	673	(18.3)	201	(33.5)	

BMI, body mass index.

^a^Analysis of Chi-square test for categorical variables.

Data are shown as a number (%).

### Validity of diabetes self-reports

Table 2 showed the validity of self-reported diabetes only using fasting plasma glucose (FPG) test. When we classified the subjects who had FPG ≥7.0 mmol/L or self-reported current use of diabetes medication as identified diabetes, the sensitivity, specificity, PPV, and NPV of self-reported diabetes were 72.0%, 99.2%, 95.1%, and 93.9%, respectively. Substantial agreement was found (Kappa = 0.78).

**Table 2.** Sensitivity and specificity analysis in validation study of self-reported type 2 diabetes in Shanghai (*n* = 4,322)

	FPG values ≥7.0 mmol/L or use of diabetes medication (*n* = 4,322)		

	Yes	No	Total
Self-reported diabetes			
Yes	578	30	608
No	225	3,489	3,714
Total	803	3,519	4,322
Validation measurements			
Sensitivity, % (95% CI)	72.0 (68.7–75.1)		
Specificity, % (95% CI)	99.2 (98.8–99.4)		
PPV, % (95% CI)	95.1 (93.0–96.7)		
NPV, % (95% CI)	93.9 (93.1–94.7)		
Kappa (95% CI)	0.78 (0.76–0.81)		

CI, confidence interval; FPG, fasting plasma glucose; NPV, negative predictive value; PPV, positive predictive value.

Data for sensitivity, specificity, PPV and NPV are shown as percentages (95% confidence intervals).

Data for Kappa is shown as Kappa value (95% confidence interval).

### Sub-group analysis of personal characteristics in validity of diabetes self-reports

We also analyzed the associations of subjects with different personal characteristics’ strength of agreement between self-reports and laboratory findings. Table 3 showed that subjects who were female, older, and had a family history of diabetes in first-degree relatives had excellent Kappa values over 0.8 and their sensitivities were all above 75%. Regardless of the gender, age, and family history of diabetes, each group had great specificity, PPV, and NPV over 90%. Table 4 showed that all validation measurements were similar among different education levels. Obesity group with BMI ≥28 had better sensitivity and PPV, but poorer specificity and NPV, than non-obesity. There was no difference in Kappa values between obese and non-obese groups.

**Table 3.** Subgroup analysis by gender, age, and family history in validation study of self-reported type 2 diabetes in Shanghai (*n* = 4,322)

	Male			Female			Age at baseline ≥60 years			Age at baseline <60 years			Have a family history of diabetes in first- degree relatives			Have no family history of diabetes in first- degree relatives		
		
	Yes	No	Total	Yes	No	Total	Yes	No	Total	Yes	No	Total	Yes	No	Total	Yes	No	Total
Self-reported diabetes																		
Yes	385	21	406	193	9	202	137	7	144	441	23	464	196	5	201	374	25	399
No	166	2,069	2,235	59	1,420	1,479	42	650	692	183	2,839	3,022	53	620	673	169	2,833	3,002
Total	551	2,090	2,641	252	1,429	1,681	179	657	836	624	2,862	3,486	249	625	874	543	2,858	3,401
Validation measurement																		
Sensitivity, % (95% CI)	69.9 (65.9–73.7)			76.6 (70.9–81.7)			76.5 (69.6–82.5)			70.7 (66.9–74.2)			78.7 (73.1–83.6)			68.9 (64.8–72.8)		
Specificity, % (95% CI)	99.0 (98.5–99.4)			99.4 (98.8–99.7)			98.9 (97.8–99.6)			99.2 (98.8–99.5)			99.2 (98.1–99.7)			99.1 (98.7–99.4)		
PPV, % (95% CI)	94.8 (92.2–96.8)			95.5 (91.7–97.9)			95.1 (90.2–98.0)			95.0 (92.7–96.8)			97.5 (94.3–99.2)			93.7 (90.9–95.9)		
NPV, % (95% CI)	92.6 (91.4–93.6)			96.0 (94.9–97.0)			93.9 (91.9–95.6)			93.9 (93.0–94.8)			92.1 (89.8–94.1)			94.4 (93.5–95.2)		
Kappa (95% CI)	0.76 (0.73–0.79)			0.83 (0.79–0.87)			0.81 (0.76–0.86)			0.78 (0.75–0.81)			0.83 (0.78–0.87)			0.76 (0.73–0.79)		

CI, confidence interval; FPG, fasting plasma glucose; NPV, negative predictive value; PPV, positive predictive value.

Data for sensitivity, specificity, PPV, and NPV are shown as percentages (95% confidence intervals).

Data for Kappa is shown as Kappa value (95% confidence interval).

**Table 4.** Subgroup analysis by education and body mass index in validation of self-reported type 2 diabetes in Shanghai (*n* = 4,322)

	High school and above			Middle school and below			BMI ≥28 kg/m^2^			BMI <28 kg/m^2^		
			
	Yes	No	Total	Yes	No	Total	Yes	No	Total	Yes	No	Total
Self-reported diabetes												
Yes	258	14	272	320	16	336	132	5	137	446	25	471
No	102	1,733	1,835	123	1,751	1,874	40	258	298	185	3,230	3,415
Total	360	1,747	2,107	443	1,767	2,210	172	263	435	631	3,255	3,886
Validation measurement												
Sensitivity, % (95% CI)	71.7 (66.7–76.3)			72.2 (67.8–76.4)			76.7 (69.7–82.8)			70.7 (67.0–74.2)		
Specificity, % (95% CI)	99.2 (98.7–99.6)			99.1 (98.5–99.5)			98.1 (95.6–99.4)			99.2 (98.9–99.5)		
PPV, % (95% CI)	94.9 (91.5–97.2)			95.2 (92.4–97.3)			96.4 (91.7–98.8)			94.7 (92.3–96.5)		
NPV, % (95% CI)	94.4 (93.3–95.5)			93.4 (92.2–94.5)			86.6 (82.2–90.2)			94.6 (93.8–95.3)		
Kappa (95% CI)	0.78 (0.75–0.82)			0.78 (0.75–0.82)			0.78 (0.71–0.84)			0.78 (0.75–0.81)		

BMI, body mass index; CI, confidence interval; FPG, fasting plasma glucose; NPV, negative predictive value; PPV, positive predictive value.

Data for sensitivity, specificity, PPV, and NPV are shown as percentages (95% confidence intervals).

Data for Kappa is shown as Kappa value (95% confidence interval).

### Validity with additional HbA1c test

We performed tests of glycated hemoglobin (HbA1c) in 708 subjects under 65 years old using a cut-off of ≥6.5% (Table 5). Although the additional HbA1c test decreased the sensitivity (62.3% vs 78.9%), NPV (93.2% vs 97.0%) and Kappa value (0.72 vs 0.85), HbA1c testing could find more potential diabetes cases compared to only using the FPG test (43 vs 19).

**Table 5.** Comparison for the validity of self-reported diabetes without or with HbA1c test (*n* = 708)

	FPG values ≥7.0 mmol/L or use of diabetes medication (*n* = 708)			FPG ≥7.0 mmol/L or HbA1c ≥6.5% or use of diabetes medication (*n* = 708)		
	
	Yes	No	Total	Yes	No	Total
Self-reported diabetes						
Yes	71	2	73	71	2	73
No	19	616	635	43	592	635
Total	90	618	708	114	594	708
Validation measurements						
Sensitivity, % (95% CI)	78.9 (69.0–86.8)			62.3 (52.7–71.2)		
Specificity, % (95% CI)	99.7 (98.8–100)			99.7 (98.8–100)		
PPV, % (95% CI)	97.3 (90.5–99.7)			97.3 (90.5–99.7)		
NPV, % (95% CI)	97.0 (95.4–98.2)			93.2 (91.0–95.1)		
Kappa (95% CI)	0.85 (0.79–0.92)			0.72 (0.65–0.80)		

CI, confidence interval; FPG, fasting plasma glucose; NPV, negative predictive value; PPV, positive predictive value.

Data for sensitivity, specificity, PPV and NPV are shown as percentages (95% confidence intervals).

Data for Kappa is shown as Kappa value (95% confidence interval).

### Analysis pre-diabetes and glycemic control

We defined self-reported diabetes as diagnosed diabetes, and no self-reported diabetes but single-visit FPG ≥7.0 mmol/L as undiagnosed diabetes. The prevalence of total diabetes in our study was 19.3% (14.1% for diagnosed diabetes and 5.2% for undiagnosed diabetes). Pre-diabetes prevalence was 26.2% when it was defined as a single-visit FPG 5.6–6.9 mmol/L. Among 608 subjects with diagnosed diabetes, the prevalence of glycemic control was 36.7%. In addition, 88.2% of diagnosed diabetes cases were taking diabetes medication currently. In 536 subjects who were taking diabetes medication currently, 36.0% controlled their FPG <7.0 mmol/L, while in 72 subjects who were not taking diabetes medication, 41.7% controlled their FPG (eTable 1).

In 708 subjects aged less than 65 years, the prevalence of diagnosed diabetes was lower than that in the validation study (10.3% vs 14.1%). An additional HbA1c test increased the prevalence of undiagnosed diabetes and pre-diabetes greatly. Using a combination of FPG and HbA1c, the prevalence of undiagnosed diabetes (defined as FPG ≥7.0 mmol/L or HbA1c ≥6.5%) was 6.1%. The prevalence of pre-diabetes reached 54.9%. Only 15.2% cases who were taking diabetes medication currently had controlled their glycemic with both FPG <7.0 mmol/L and HbA1c <6.5% (eTable 2).

## DISCUSSION

In this validation study, the sensitivity, specificity, PPV, and NPV of self-reported diabetes were 72.0%, 99.2%, 95.1% and 93.9%, respectively. The Kappa value was 0.78. Additional HbA1c testing may result in better specificity and PPV but poorer sensitivity, NPV, and Kappa coefficient. The subgroups of women, those aged over 60 years, or those having a family history of diabetes had better agreement. The prevalence of type 2 diabetes was 19.3% in our validation study, including 14.1% diagnosed diabetes and 5.2% undiagnosed diabetes. Pre-diabetes was observed in 26.2% of subjects. The results from subjects with additional HbA1c test indicated an increased prevalence of undiagnosed diabetes and pre-diabetes.

The prevalence of diabetes has increased significantly in recent decades in China. It was less than 1% in the Chinese population in 1980.^28^ In subsequent national surveys conducted in 1994, 2000, 2007, and 2010, the prevalence proportions of diabetes were 2.5%, 5.5%, 9.7%, and 11.6%, respectively.^29^^–^^32^ The most recent national survey in 2013 reported that the prevalence of diabetes was 10.9%, but in Han participants, the prevalence was 14.7%.^33^ For the same age groups in our study, the national prevalence of self-reported diabetes is 7.0%.^32^ As mentioned, urban residents and economically developed regions have a high prevalence in mainland China.^32^ Our study was conducted in Shanghai, one of the most developed cities in China. All participants were living in the urban area. The prevalence of 19.3% in our study is higher than the national statistic of 14.7% in 2013.

The reliability, validity, and consistency of self-reported information and criterion standards, such as health status and the medical record, is critical for health researchers. There are a number of studies that have shown high rates of confirmation of self-reported diabetes diagnosis based on information on medication or other data from medical record.^13^^,^^17^^,^^21^ Validation of medical record review could not directly provide sensitivity or specificity of medical history because it is impossible to screen records systematically for those subjects who did not report any medical history.^15^ By contrast, some studies used laboratory assays to validate self-reported diabetes.^6^^,^^9^^,^^10^^,^^14^ Our results are consistent with those of prior studies reporting specificity and sensitivity for prevalent self-reported diabetes compared with the same definition (FPG ≥7.0 mmol/L or diabetes medication use).^14^ The sensitivity in our study is higher than that in other studies validated using laboratory assays with both FPG and HbA1c.^6^^,^^9^^,^^10^^,^^14^^,^^15^ This can be partly explained by the fact the undiagnosed subjects will remain hidden because we only use FPG as the screening test. As expected, the specificity over 99% in this study was markedly high, which is consistent with the findings of previous studies that used laboratory assays.^6^^,^^10^^,^^14^^,^^15^ The Kappa agreement in our study was 0.78, which was closed to other studies conducted in developed countries.^7^^,^^11^^,^^15^ Overall, we found self-reported data on diabetes were more specific than sensitive; therefore, an underestimation of prevalence and attenuation of associations with risk factors can be expected.^10^ Previous assays suggested that self-reports of diabetes may have relatively low sensitivity and high specificity. But if laboratory assays are hard to obtain, self-report can be useful for identifying diabetic individuals.^6^ As that validated by laboratory findings in the Saku study, the specificity of self-reported diabetes in our study was fairly high, a finding which supports the use of self-reported diabetes as a measure of diabetes status.^6^ With almost perfect specificity, the non-differential misclassification bias due to self-reported diabetes would have little impact relative risk measures.

Because of well-defined diagnostic criteria and the requirement for ongoing disease management, self-reported diabetes has higher agreements and important values for epidemiologic studies and surveys than many other chronic conditions.^7^ But the accuracy of self-reported data on medical history depends on the subjects’ knowledge and understanding of the relevant information, ability to recall it, willingness to report and the diagnosis of disease status.^10^^,^^34^ The rate of incorrect reporting and misclassification can be significant and different by population, disease, and the severity of the disease.^10^^,^^35^^,^^36^ Reliability of reporting can also be influenced by personal characteristics, such as age, gender, and culture. Okura et al reported that the agreement was enhanced with younger age, female sex, and high education level.^8^^,^^11^ In the Saku cohort study, the agreement was slightly higher among women than among men.^6^ Those with higher BMI were more likely to accurately self-report diabetes in the Women’s Health Initiative study.^4^ Our study shows that subjects of female sex, older age, and those having a family history of diabetes in first-degree relatives had better agreement. Recently, annual health checkups provided by local government in the older population aged over 65 years may increase the awareness of diabetes in study communities. It also makes the older group have better Kappa value than younger subjects. Having a family history of diabetes as the risk factor of diabetes is accepted in common people. Subjects with that characteristic might pay closer attention to diabetes, which leads to better sensitivity and PPV of self-reported disease. These characteristic factors need to be taken into account when interpreting self-administered questionnaire data on certain diseases. The results of different self-report validity among participants with different characteristics will also provide scientific evidence to guide health care organization for health promotion in different population.

The resources are often used in the validation study of self-reported diabetes in epidemiologic studies or surveys, including medical records and tests of HbA1c. Although record linkage to medical record alone has a better agreement than laboratory assay alone, it does not mean that medical record review is better than laboratory assay. Medical record review could find missed diabetes cases that the subjects may not be able to recall the diagnosis reported by the physician and also misunderstand or be unwilling to report it, but could not find undiagnosed diabetes. On the other hand, using only medical record review may lead to the underestimation of the association between diabetes and other diseases risk. Total diabetes included both previously diagnosed diabetes and previously undiagnosed diabetes.^31^ In the world, 49.7% of adults with diabetes are undiagnosed. This situation varies in different regions, from 37.6% to 69.2%.^1^ Among the Chinese general population, only 36.5% of diabetes patients were aware of their disease,^33^ while the proportion was over 60% in our validation study. Living in an economically developed area and having good health care should be the main causes to improve awareness in our cohort. The data of the National Health and Nutrition Examination Survey (NHANES) indicated that the HbA1c cut-points identify fewer cases of undiagnosed diabetes or pre-diabetes than the FPG cut-points.^37^ In the China Health and Retirement Longitudinal Study (CHARLS), among persons aged 45 years and above, 17.4% have diabetes and another 41.9% have pre-diabetes. Among people with diabetes, 59.3% are currently undiagnosed.^38^ The higher prevalence of diabetes and pre-diabetes for the middle aged and the elderly persons in our study is in accordance with the result from CHARLS that suggest the problem is worse among those who lived in urban coastal areas.^38^ In our study, the rate of taking diabetes medication in diagnosed cases was similar to another Chinese survey, in which the prevalence proportions were 85–90%. In addition, these surveys also had similar glycemic control, of which the prevalence proportions were 35–40%.^32^^,^^38^^,^^39^ Our data showed that control of both HbA1C and FPG was difficult.

The strengths of this study included its large sample size and use of laboratory assays for FPG and HbA1c levels. Diabetic medication is used daily over the long term. We limited the recall time to the preceding 24 hours to minimize memory bias. So the probability of errors for misclassification in our study could be smaller than in studies with longer recall. We also considered participant’s characteristics, such as gender, age, education, obesity, and family history of diabetes in first-degree relatives, when assessing and interpreting the validation of self-reported diabetes. This validation study will provide an advantage for further research in the epidemiological studies of type 2 diabetes. Despite its strengths, our study has some limitations. First, because we did not perform a medical record review, that may have underestimated the number of true positives and overestimated the number of true negatives, which might have biased our estimates. Second, our results reflect the finding only for subjects who came to the checkup without randomly selecting from these two cohorts. Third, although OGTT is not a necessary test for diabetes diagnose, the lack of OGTT data limits interpretability of results regarding undiagnosed DM and impaired glucose tolerance.

In conclusion, this validation study suggests that self-reported diabetes is a valid measure of diabetes in the general population in urban Shanghai. Participants with different personal characteristics may have different indicators in terms of validation, such as gender, age, and family history of diabetes in first-degree relatives. There were no differences in agreement in different education or obesity groups. Our findings indicate that self-reported questions for diabetes in the questionnaire were generally accurate and a reliable tool in large, population-based epidemiologic studies.

## APPENDIX A. SUPPLEMENTARY DATA

eTable 1. Prevalence of diabetes, pre-diabetes, and glycemic control among all subjects using only FPG test (n = 4,322)

eTable 2. Prevalence of diabetes, pre-diabetes, and glycemic control among subjects using both FPG and HbA1c test (n = 708)
