# Supplementary material for: Personal Characteristics Effects on Validation of Self-reported Type 2 Diabetes From a Cross-sectional Survey Among Chinese Adults
Source: J Epidemiol. 2020 Nov 5;30(11):516–21. doi: 10.2188/jea.JE20190178 (PMC7557172; doi:10.2188/jea.JE20190178)
Supplement: Supplementary file 1 [file je-30-516-s001.pdf]

Supplementary Information

Personal characteristics effects on validation of self-reported type 2 diabetes from a cross-sectional survey among Chinese adults

Hong-Lan Li, Jie Fang, Long-Gang Zhao, Da-Ke Liu, Jing Wang, Li-Hua Han, Yong-Bing Xiang*

eTable 1. Prevalence of diabetes, pre-diabetes, and glycemic control among all subjects using only FPG test (n=4,322)

eTable 2. Prevalence of diabetes, pre-diabetes, and glycemic control among subjects using both FPG and HbA1c test (n=708)

eTable 1. Prevalence of diabetes, pre-diabetes, and glycemic control among all subjects using only FPG test (n=4,322)

	number	%
Prevalence of diabetes		
Diagnosed diabetes (self-reported diabetes)	608	14.1
Undiagnosed diabetes (self-reported no diabetes & FPG ≥7.0 mmol/L)	225	5.2
Total diabetes (diagnosed & undiagnosed diabetes)	833	19.3
Pre-diabetes		
Pre-diabetes (self-reported no diabetes & 5.6≤FPG<7.0 mmol/L)	1,133	26.2
Glycemic control		
Currently taking diabetes medication in diagnosed diabetes	536	88.2
FPG <7.0 mmol/L in diagnosed diabetes	223	36.7
FPG <7.0 mmol/L in diagnosed diabetes with currently taking diabetes medication	193	36.0
FPG <7.0 mmol/L in diagnosed diabetes but no taking diabetes medication	30	41.7

FPG, fasting plasma glucose.

eTable 2. Prevalence of diabetes, pre-diabetes, and glycemic control among subjects using both FPG and HbA1c test (n=708)

	number	%
Prevalence of diabetes		
Diagnosed diabetes (self-reported diabetes)	73	10.3
Undiagnosed diabetes 1 (self-reported no diabetes & FPG ≥7.0 mmol/L)	19	2.7
Undiagnosed diabetes 2 (self-reported no diabetes & HbA1c ≥6.5%)	40	5.6
Undiagnosed diabetes 3 (self-reported no diabetes & 'FPG ≥7.0 mmol/L or HbA1c ≥6.5%')	43	6.1
Total diabetes (diagnosed & undiagnosed diabetes 3)	116	16.4
Pre-diabetes		
Pre-diabetes 1 (self-reported no diabetes & 5.6≤FPG<7.0 mmol/L)	134	18.9
Pre-diabetes 2 (self-reported no diabetes & 5.7%≤HbA1c<6.5%)	353	49.9
Pre-diabetes 3 (self-reported no diabetes & '5.6≤FPG<7.0 mmol/L or 5.7%≤HbA1c<6.5%')	389	54.9
Glycemic control		
Currently taking diabetes medication in diagnosed diabetes	66	90.4
FPG <7.0 mmol/L & HbA1c <6.5% in diagnosed diabetes	12	16.4
FPG <7.0 mmol/L & HbA1c <6.5% in diagnosed diabetes with currently taking diabetes medication	10	15.2
FPG <7.0 mmol/L & HbA1c <6.5% in diagnosed diabetes but no taking diabetes medication	2	28.6

FPG, fasting plasma glucose; HbA1c, glycated hemoglobin.
